# Drought‐stress induced changes of fatty acid composition affecting sunflower grain yield and oil quality

**DOI:** 10.1002/fsn3.3690

**Published:** 2023-09-19

**Authors:** Mehdi Ghaffari, Amir Gholizadeh, Saeed Rauf, Farnaz Shariati

**Affiliations:** ^1^ Oil Crops Research Department, Seed and Plant Improvement Institute Agricultural Research Education and Extension Organization (AREEO) Karaj Iran; ^2^ Crop and Horticultural Science Research Department, Golestan Agricultural and Natural Resources Research and Education Center Agricultural Research Education and Extension Organization (AREEO) Gorgan Iran; ^3^ Department of Plant Breeding and Genetics, College of Agriculture University of Sargodha Sargodha Pakistan

**Keywords:** drought stress, grain yield, linoleic acid, oil quality, sunflower

## Abstract

Water availability is the most important key factor affecting sunflower productivity and its oil quality. This study investigated the effect of drought stress on sunflower fatty acids and its effects on grain yield and related components. Thirteen sunflower hybrids were evaluated as randomized complete block design with three replications under normal and drought stress conditions in Karaj, Iran, during 2 years (2019 and 2020). Drought stress was imposed by water withholding during the reproductive stage. Drought stress accelerated the maturity of sunflower and caused a reduction in grain yield (30%), grains weight (11%), and grain numbers/head (22%) compared with normal irrigation. Means of grain yield were 2.7 and 1.8 t/ha under normal and drought stress conditions respectively. Grain numbers/head had higher correlation with grain yield than grains weight under both conditions. Among the fatty acids, the contents of palmitic and linoleic acids were increased (11% and 3%, respectively) while stearic and oleic acids were decreased (6% and 11%). The results indicated that sunflower hybrids benefit from the escape strategy differentially to adapt drought stress condition. However, this adaptation changes sunflower fatty acid profile that reduces grain yield and quality of sunflower oil in Karaj conditions in Iran. In order to achieve the higher yields and higher oil quality, it is necessary to avoid drought stress in sunflower production fields.

## Graphical abstract

Drought stress can affect agronomic and quality characteristics of sunflower. The results indicated that sunflower hybrids benefit from the escape strategy differentially to adapt to the drought stress condition. However, this adaptation changes sunflower fatty acid profile that reduces grain yield and quality of sunflower oil. In order to achieve higher yields and higher oil quality in sunflower, cultivation of standard oil sunflower hybrids and providing full water requirement is necessary.

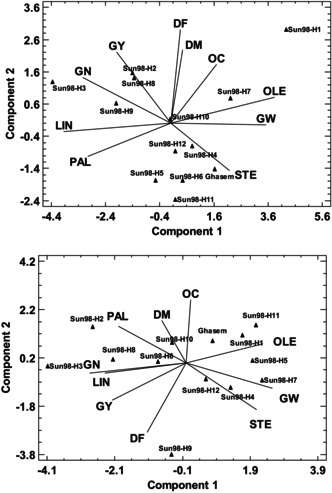

## 1 INTRODUCTION

Sunflower (*Helianthus annuus* L.) is one of the main vegetable oil sources with an annual production area of about 27 million hectares worldwide (FAO, 2019). Sunflower, the first confectionary type, was introduced to Iran, coincided with World War I. Cultivation of oil‐type sunflower has been started in 1965 and increased to more than 100,000 ha in the early 1990s (Ghaffari et al., 2020). Sunflower breeding in Iran has been implemented since 1969 with remarkable achievements in producing sunflower single cross hybrids (Ghaffari et al., 2019). Although sunflower is moderately tolerant to drought stress, its productivity is affected by drought stress considerably (Chimenti et al., 2002). Numerous studies express grain yield and oil content of sunflower as the main sensitive parameters to drought stress during flowering and reproductive stages (Akcay & Dagdelen, 2016; Ghaffari et al., 2012; Todorovic et al., 2009).

The sunflower oil is desirable for human consumption due to its favorable fatty acid composition (Baydar & Erbaş, 2005). Sunflower oil quality is affected by seed oil content and fatty acid composition of the oil and defines the oil's value for industry (Rondanini et al., 2003). The fatty acid composition of sunflower determines its uses and health advantages on human beings, while oil content determines its economic value (Zheljazkov et al., 2009).

For oil quality purposes, oleic and linoleic acids are the most important fatty acids because these contribute almost 90% to the total fatty acid content in sunflower oil (Van Der Merwe et al., 2013). The amount of oleic acid determines the quality of sunflower oil more than other fatty acids because it is important in both edible purposes and biodiesel production (Mensink et al., 2003). Standard sunflower oil, on average, consists mainly of about 69% linoleic (C18:2), 20% oleic (C18:1), 7% palmitic (C16:0), and 4% stearic (C18:0) fatty acids (Skoric et al., 2008). Other fatty acids including arachidic (20:0), behenic (22:0), and lignoceric (24:0) acids constitute a minor part of sunflower oil (Friedt et al., 1994).

The genotype is the most important factor that determines the fatty acid composition of an oil (Petcu et al., 2001), but also environmental factors such as water availability and fluctuations in temperature during the grain‐filling stage can widely affect the oil percentage and fatty acid composition of oil (Anastasi et al., 2010; Izquierdo et al., 2002).

Research studies are looking into the effects of climatic conditions on fatty acid composition of sunflower oil. Zheljazkov et al. (2009) demonstrated that later planting tended to increase total saturated fatty acids, mainly palmitic and stearic acids, in sunflower at five locations in Mississippi. Lajara et al. (1990) reported that geographic location significantly affects the fatty acid composition of sunflower in Spain. Weiss (2000) found that seeds maturing at higher temperatures accumulate higher oil content. Demurin et al. (2000) reported that oleic acid content of sunflower is affected by temperature during grain development, and increasing each unit of temperature in C° leads to about two percent increase in oleic acid content. Erdemoglu et al. (2003) reported that genotypes and climatic conditions, such as temperature, altitude, and soil structure, affect the oil content of sunflower more than irrigation. There are reports showing that the ratio of oleic to linoleic acid decrease under early‐sowing conditions (Flagella et al., 2002). Studies by Vick et al. (2004) demonstrated that environmental conditions significantly affect the relative proportion of total saturated fatty acids in sunflower. Izquierdo and Aguirrezábal (2008) detected that increased temperature may increase the concentration of behenic acid in sunflower oil. The oleic/linoleic acids ratio increases at higher temperatures occurring during seed maturation and, on the contrary, decreases at lower temperatures (Sukkasem et al., 2013).

Information on the affectability of the fatty acid profile of sunflower by drought stress is limited. Petcu et al. (2001) reported reduction of oleic acid but increasing of linoleic acid contents in sunflower under water‐stressed condition. Baldini et al. (2000) reported that water stress leads to a reduction in oleic acid in standard but an increase in high oleic sunflower hybrids. They also noted that saturated fatty acids, such as palmitic and stearic acids, did not vary concerning the water regime. Ali et al. (2009) reported that sunflower genotypes express differential fatty acids profile in response to water stress. Popa et al. (2017) reported a significant negative effect of drought conditions on oleic acid concentration during seed maturation in all of the studied sunflower hybrids. Delayed planting also decreased the concentration of oleic acid and increased linoleic acid concentration. They concluded that rainfall, genotype, and planting date influenced yield and fatty acid composition in sunflower.

Although abundant literature is available on sunflower response to drought stress in terms of agronomical characteristics, little has been done to study the effects of drought stress on fatty acid composition in this oil crop. The present study aimed to provide information about fatty acid variability of sunflower oil under drought stress and the affectability of sunflower grain yield by these changes in Karaj, Iran.

## 2 MATERIALS AND METHODS

**FIGURE 1:**
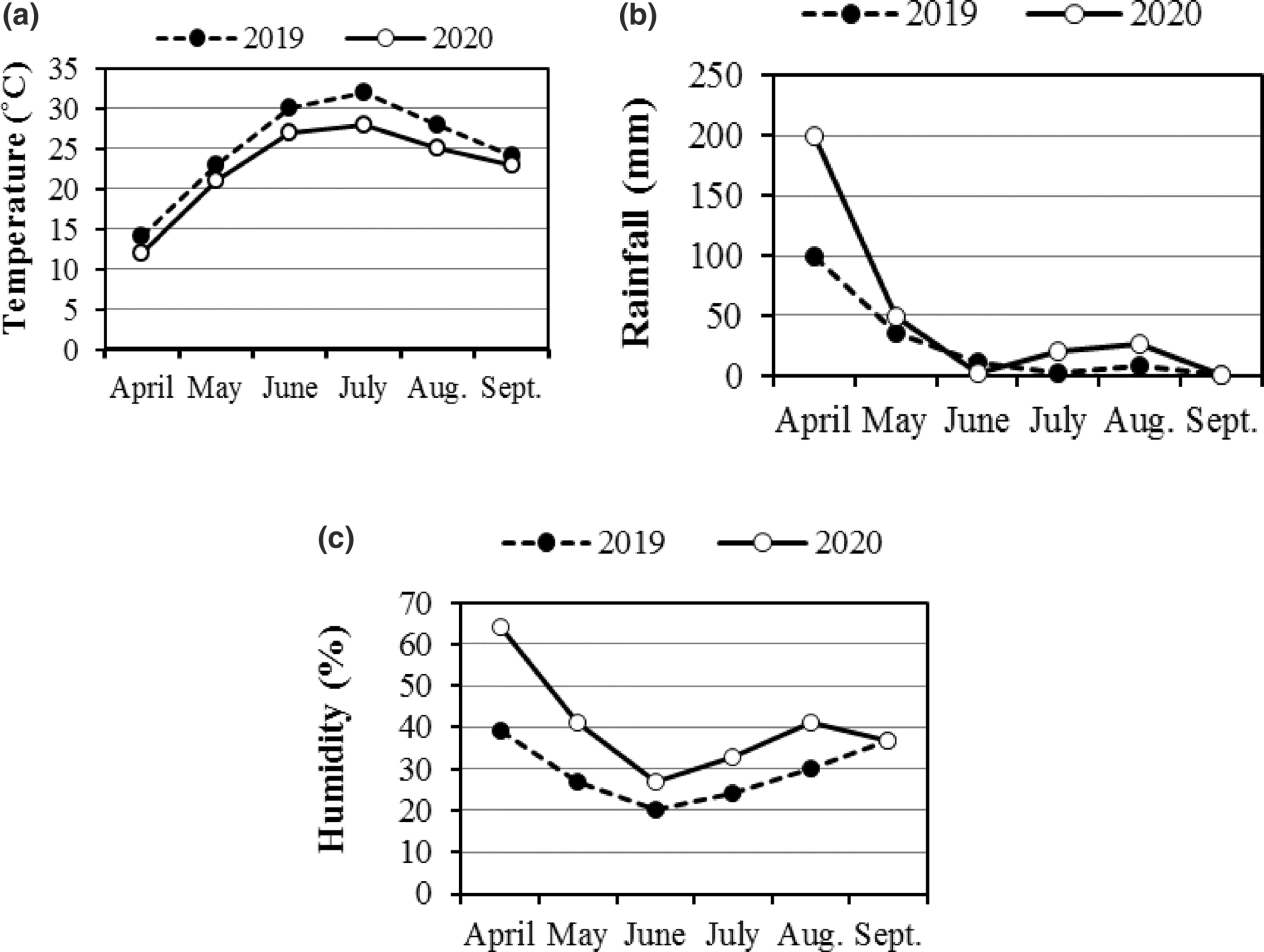
Average monthly data for temperature (a), rainfall (b) and humidity (c) in Karaj, Iran for growing seasons in 2019 and 2020.

The field study was conducted at the research field of Seed and Plant Improvement Institute (SPII) in Karaj, Iran (35.84° N, 50.93° E; altitude of 1321 m above sea level) during 2 years (2019 and 2020). The region has a Mediterranean climate with hot dry summers and cold dry winters with average annual precipitation of 243 mm and annual temperature of 13.5°C. The monthly average climatological data for the 2 years of study is represented in Figure 1. Physicochemical characteristics of soil in the experimental field were given in Table 1.

**TABLE 1:** Average physicochemical characteristics of soil in the experimental field.

Electrical conductivity (dS/m)	pH	Organic carbon (mg/kg)	N (mg/kg)	P (mg/kg)
2.2	7.2	5800	600	12.6

K (mg/kg)	Clay (%)	Silt (%)	Sand (%)	Soil texture
256	26	49	25	Clay Loam

A set of 13 sunflower hybrids (Table S1) was evaluated in a randomized complete block design with three replications under normal and drought stress conditions. Drought stress was imposed by water withholding in the R1‐R6 growth stages as defined by Schneiter and Miller (1981). Each experimental plot consisted of three rows of 4 m in length with 60 and 25 cm spacing between and within rows, respectively. Fertilizers were applied at the rate of 100:70:90 kg/ha for N:P:K. All phosphate and potassium fertilizers and one‐third of nitrogen fertilizer were distributed before planting, and the rest of the nitrogen fertilizer was applied in two splits up to 8–10 leaf stage. Grain yield and oil characteristics were recorded following the harvest at physiological maturity (R9). Oil content was determined using a Soxhlet extractor. In order to determine the profile of fatty acids initially, fatty acid methyl esters (FAMEs) were prepared and then FAMEs analyzed by gas chromatograph as follows: The required amount of oil was extracted using 30 g of seed sample and by Soxhlet method. FAMEs were prepared from the oil samples according to the method reported by Savage et al. (1997). Concisely, 2 mL of NaOH (0.01 M in methanol) was added to a tube containing the oil sample (ca. 10 mg) dissolved in 0.5 mL hexane and then held in a water bath at 60°C for 10 min. After that, 3 mL boron trifluoride (BF3) (20% in methanol) was added to the sample tube and it was placed in a 60°C water bath. After 10 min, the tube was cooled under running cold water and then 2 mL of sodium chloride (20%, weight/volume) and 1 mL of hexane were added. After complete mixing and centrifugation, the upper hexane layer containing FAMEs was ready to inject to gas chromatograph. FAMEs were analyzed by a gas chromatograph (Varian, CP‐3800) with a flame ionization detector and a split/split‐less injector. A fused‐silica capillary column (CP SIL 88 for FAME; 50 m × 0.25 mm, 0.2 μm film thickness) was used for analysis. The temperature of the injector and detector was 270°C and 300°C, respectively, and the oven temperature was 190°C. Carrier gas was nitrogen. The FAMEs were identified by comparison of their retention times with standard FAMEs, and peak area was reported as a percentage of the total fatty acids.

Combined analyses of variance followed by means comparison were performed for agronomic data and for the main fatty acids – palmitic, stearic, oleic and linoleic acids – using SAS (Ver. 9.2) software. The mean comparison was based on the highest significant levels for interaction effects. Stress susceptibility index (SSI) (Fischer & Maurer, 1978) and stress tolerance index (STI) (Fernandez, 1992) were calculated to draw three‐dimensional plots of grain yield under normal (GYn) and drought stress conditions. Principal component analysis (PCA) (Jolliffe, 2002) was used to study the relationships between the traits and the hybrids using Stat‐graphics (Ver. 16.1.11, 2007).

## 3 RESULTS

### 3.1 Analysis of variance

According to the combined analysis of variance, the years significantly affected flowering time (*p* = .003), 1000‐grains weight (*p* = .003), and all of the fatty acids (*p* ≤ .001) (Table 2). The effect of irrigation treatments was significant only for oleic acid content (*p* = .001). There were significant interaction effects over the years by irrigation treatments for oil content (*p* = .022) and oleic and linoleic acids (*p* ≤ .0001). Due to the significant differences between the hybrids (*p* ≤ .003) in terms of all of the measured traits, there was considerable genetic variability between the hybrids. The interaction effects of the hybrids with the years were significant for all of the traits (*p* ≤ .019–.001) except for flowering time (*p* = .056). Significant interaction effects of hybrids by irrigation treatments were observed only for days to flowering (*p* = .001) and days to maturity (*p* = .013). Three‐way interactions of hybrids × year × irrigation treatments were significant for grain yield (*p* = .018), oil content, and all of the fatty acid components at *p* = .0001.

**TABLE 2:** Combined analysis of variances for agronomic and oil characteristics of sunflower under normal and drought stress conditions in 2 years (2019 and 2020).

SOV	DF	Days to flowering	Days to maturity	1000‐grains weight	Grains no./head	Grain yield
Year (Y)	1	83.31**	7.41ns	525.07**	21,077.29ns	0.36ns
Irrigation (I)	1	509.77ns	715.10ns	1140.48ns	1,384,758.88ns	25.34ns
Y × I	1	5.03ns	6.56ns	68.54ns	52,649.89 ns	0.21ns
R (Y × I)	8	4.51	31.18	28.42	12,361.31	0.18
Hybrid (H)	12	34.46**	30.08**	147.21**	169,462.06**	0.39**
H × Y	12	1.43ns	2.42*	97.86*	27,846.34**	0.25**
H × I	12	8.89**	1.59**	55.00ns	23,530.15ns	0.85ns
H × Y × I	12	0.84ns	0.41ns	47.48ns	12,894.84ns	0.17*
Error	96	0.79	1.11	42.90	8891.88	0.79
Coefficient of variations (%)		1.55	1.07	13.46	12.18	12.49

SOV	DF	Oil content	Palmitic acid	Stearic acid	Oleic acid	Linoleic acid
Year (Y)	1	8.12ns	13.31**	2.00**	166.65**	132.83**
Irrigation (I)	1	247.34ns	16.71ns	4.25ns	237.77**	188.42ns
Y × I	1	61.88*	2.12**	4.35**	0.00ns	9.20ns
R (Y × I)	8	7.76	0.03	0.08	1.93	2.84
Hybrid (H)	12	10.38**	2.36**	5.48**	111.95**	115.70**
H × Y	12	7.03**	0.82**	3.80**	31.66**	34.96**
H × I	12	9.06ns	1.03ns	0.52ns	6.02ns	9.09ns
H × Y × I	12	6.65**	0.78**	1.31**	5.04**	8.93**
Error	96	1.56	0.06	0.03	0.74	1.05
Coefficient of variations (%)		2.99	3.76	3.05	4.17	1.54

*Note*: ns, * and ** denote the non‐significant and significant differences at (*α* = .05) and (*α* = .01), respectively.

Abbreviations: DF, degrees of freedom; SOV, sources of variation.

### 3.2 Effect of drought stress on agronomic traits

The hybrids started to flowering in a range of 56–62 days under normal and in a range of 53–57 days under drought stress condition. The range of days to maturity was 98–104 days and 95–99 days under normal and drought stress conditions, respectively. So, flowering date was reduced in a range of 2–6 days, and days to maturity in a range of 3–5 days indicated a differential response of the hybrids to drought stress (Table 3).

**TABLE 3:** Mean of the sunflower hybrids for phenological characteristics under normal (N) and drought stress (S) conditions and grain yield components during 2 years (2019 and 2020).

Hybrids	Days to flowering		Days to maturity		1000‐grains weight		Grain numbers/head	
	N	S	N	S	2019	2020	2019	2020
Sun98‐H1	62.5 ± 0.4	56.2 ± 0.8	103.8 ± 0.4	98.8 ± 0.7	46.5 ± 4.9	58.2 ± 4.9	761.2 ± 42.4	695.7 ± 51.5
Sun98‐H2	60.7 ± 0.6	55.2 ± 0.8	103.0 ± 0.4	97.7 ± 1.1	40.0 ± 2.1	52.9 ± 3.6	873.5 ± 33.9	819.2 ± 52.1
Sun98‐H3	59.3 ± 0.4	57.3 ± 0.4	102.2 ± 0.7	97.0 ± 1.1	41.8 ± 3.0	36.0 ± 2.8	1076.0 ± 92.1	1064.5 ± 64.5
Sun98‐H4	57.5 ± 0.3	55.0 ± 0.6	101.2 ± 0.7	96.7 ± 0.6	47.1 ± 2.6	53.1 ± 2.2	847.0 ± 27.6	672.5 ± 45.1
Sun98‐H5	56.5 ± 0.4	54.5 ± 0.8	99.5 ± 0.4	95.7 ± 0.7	42.9 ± 1.3	53.0 ± 1.0	700.7 ± 57.9	655.5 ± 66.2
Sun98‐H6	56.7 ± 0.4	55.0 ± 0.4	99.7 ± 0.5	96.0 ± 0.5	49.4 ± 2.3	53.4 ± 1.3	728.2 ± 40.7	632.7 ± 25.5
Sun98‐H7	61.5 ± 0.4	55.5 ± 0.3	100.8 ± 0.5	97.2 ± 0.9	53.8 ± 1.4	52.3 ± 3.2	653.2 ± 62.0	713.7 ± 52.7
Sun98‐H8	61.0 ± 0.5	56.0 ± 0.7	102.8 ± 0.3	98.3 ± 1.0	47.0 ± 2.3	48.5 ± 2.2	817.3 ± 31.1	915.2 ± 52.4
Sun98‐H9	62.5 ± 0.2	58.7 ± 0.6	98.3 ± 0.4	95.0 ± 0.7	46.9 ± 2.2	49.2 ± 4.0	885.0 ± 78.0	889.3 ± 104.0
Sun98‐H10	60.3 ± 0.5	55.5 ± 0.8	103.0 ± 0.4	97.7 ± 1.2	50.3 ± 4.3	47.5 ± 2.6	666.2 ± 29.6	815.2 ± 80.9
Sun98‐H11	56.3 ± 0.6	53.2 ± 0.3	99.5 ± 0.4	95.5 ± 0.7	46.3 ± 3.4	53.6 ± 1.6	765.0 ± 88.0	589.5 ± 59.6
Sun98‐H12	57.8 ± 0.4	56.2 ± 0.3	98.7 ± 0.4	95.2 ± 0.9	47.5 ± 3.4	49.0 ± 2.5	774.8 ± 74.1	818.0 ± 66.2
Ghasem	57.2 ± 0.5	54.7 ± 0.7	99.0 ± 0.6	95.2 ± 1.1	49.2 ± 3.1	49.6 ± 3.4	666.0 ± 23.3	631.0 ± 63.9
Mean	59.2	55.6	100.9	96.6	46.8	50.5	785.7	762.4
LSD 1%	1.6	1.8	1.3	1.3	8.51	8.2	217.7	239.4
C (%)	−6.1		−4.3		7.9		−3.0	

*Note*: Values are means ± Standard error.

Abbreviation: C, Changes under drought stress condition compared with normal irrigation for days to flowering and maturity and changes in 2020 compared with 2019 for yield components.

Grain yield was reduced by drought stress by 0.7 (28%) and 0.9 t/ha (32%) in 2019 and 2020, respectively. The mean values for grain yield in 2020 was higher than 2019 in both normal (2.7 t/ha) and drought stress conditions (1.9 t/ha) (Table 4). In 2020, Sun98‐H2 had the highest grain yield (3.3 t/ha) under normal irrigation followed by Sun98‐H8 (3.1 t/ha) with the highest grain yield (2.2 t/ha) under stressed condition. In 2019, Sun98‐H3 was the high‐yielding hybrid under normal (3.1 t/ha) and stressed (2.2 t/ha) conditions.

**TABLE 4:** Comparison of grain yield and oil content of sunflower hybrids under normal (N) and drought stress (S) conditions.

Hybrids	Grain yield (t/ha)				Oil content (%)			
	2019		2020		2019		2020	
	N	S	N	S	N	S	N	S
Sun98‐H1	2.6 ± 0.1	1.6 ± 0.1	2.7 ± 0.4	2.1 ± 0.1	44.9 ± 0.9	40.2 ± 0.8	44.9 ± 1.70	42.0 ± 0.4
Sun98‐H2	2.2 ± 0.1	1.9 ± 0.1	3.3 ± 0.3	1.9 ± 0.1	41.9 ± 0.8	40.1 ± 1.2	45.4 ± 0.58	42.4 ± 0.4
Sun98‐H3	3.1 ± 0.1	2.2 ± 0.2	2.8 ± 0.2	1.8 ± 0.1	42.6 ± 1.4	39.7 ± 0.7	43.7 ± 1.45	40.5 ± 1.0
Sun98‐H4	2.8 ± 0.1	2.0 ± 0.1	2.5 ± 0.2	1.8 ± 0.1	41.6 ± 0.1	38.2 ± 0.2	44.0 ± 0.67	39.8 ± 0.4
Sun98‐H5	2.0 ± 0.1	1.5 ± 0.1	2.6 ± 0.2	1.6 ± 0.1	43.1 ± 0.7	40.3 ± 0.7	41.2 ± 0.67	39.9 ± 0.3
Sun98‐H6	2.5 ± 0.2	1.8 ± 0.1	2.0 ± 0.1	2.0 ± 0.1	43.7 ± 2.3	37.8 ± 0.3	43.5 ± 0.67	41.0 ± 0.9
Sun98‐H7	2.4 ± 0.1	1.7 ± 0.1	2.6 ± 0.3	1.9 ± 0.1	43.8 ± 0.5	38.1 ± 0.5	44.4 ± 0.67	39.8 ± 0.2
Sun98‐H8	2.5 ± 0.1	2.1 ± 0.1	3.1 ± 0.2	2.2 ± 0.1	44.3 ± 0.8	39.0 ± 0.8	42.1 ± 0.67	41.5 ± 0.5
Sun98‐H9	3.1 ± 0.2	2.0 ± 0.1	2.9 ± 0.1	2.1 ± 0.1	45.1 ± 1.0	36.3 ± 0.2	39.5 ± 0.67	40.7 ± 0.9
Sun98‐H10	2.4 ± 0.1	1.6 ± 0.2	3.0 ± 0.1	1.7 ± 0.2	42.0 ± 0.1	40.2 ± 1.1	41.4 ± 0.67	41.4 ± 0.7
Sun98‐H11	2.7 ± 0.1	1.6 ± 0.3	2.2 ± 0.3	1.6 ± 0.1	42.8 ± 0.3	41.5 ± 0.8	38.6 ± 0.67	41.6 ± 0.8
Sun98‐H12	2.5 ± 0.1	1.8 ± 0.1	2.9 ± 0.2	1.9 ± 0.1	42.4 ± 1.4	39.2 ± 0.7	42.0 ± 0.33	43.3 ± 1.1
Ghasem	2.4 ± 0.1	1.9 ± 0.3	2.7 ± 0.0	1.6 ± 0.4	45.7 ± 0.6	44.1 ± 0.6	42.6 ± 0.41	43.2 ± 0.3
Mean	2.6	1.8	2.7	1.9	43.4	39.6	42.6	41.3
LSD 1%	0.6	0.5	0.6	0.4	3.9	1.8	2.7	2.6
C (%)	−30.8		−29.6		−8.8		−3.1	

*Note*: Values are means ± Standard error.

Abbreviation: C, Changes under stressed condition in compared with normal irrigation.

The mean of 1000‐grains weight was increased by about 4 g in 2020. The highest weight was observed for Sun98‐H1 (58.2 g) in 2020 (Table 3). The highest increase in grains weight was recorded for Sun98‐H2 (13 g). The mean number of grains per head decreased by 23 g in the second year; however, there were differentiating responses of the hybrids to the years. The highest reduction in grain numbers was observed in Sun98‐H11 (176 grains), while the highest increase was observed in Sun98‐H10 (149 grains).

### 3.3 Effect of drought stress on oil characteristics

Regarding the significance of the three‐way interaction effect, the highest oil content was recorded for the check hybrid, Ghasem under both normal (45.7%) and stressed (44.1%) conditions in 2019, but in 2020 Sun98‐H2 and Sun98‐H12 had the highest oil content under optimum and drought stress conditions (45.4% and 43.3%, respectively) (Table 4). The oil content was decreased under drought conditions in both years; however, the amount of reduction was greater in 2019 (3.8% and 1.3% in 2019 and 2020, respectively). The highest reduction of oil content was observed in Sun98‐H9 by about 9% in 2019.

There was a general increase in palmitic acid content under drought stress in both years; 0.89% in 2019 and 0.42% in 2020. However, differentiated responses were observed between the hybrids (Table 5). The highest palmitic acid content was recorded for Sun98‐H6 (8.76%) under the stressed condition in 2019. The maximum content of this fatty acid under normal irrigation was recorded for Sun98‐H2 (7.05%) in 2019. The content of this fatty acid was higher in 2019 than in 2020 under both normal (6.20%) and stressed (7.09%) conditions. The lowest palmitic acid content was observed in Sun98‐H1 under normal irrigation in 2020 (4.98%). The hybrid Ghasem had the lowest palmitic acid content under drought stress condition in 2020 (5.31%).

**TABLE 5:** Comparison of sunflower hybrids for palmitic and stearic acids contents (%) under normal (N) and drought stress (S) conditions.

Hybrids	Palmitic acid				Stearic acid			
	2019		2020		2019		2020	
	N	S	N	S	N	S	N	S
Sun98‐H1	5.20 ± 0.02	6.91 ± 0.04	4.98 ± 0.18	6.08 ± 0.14	5.41 ± 0.13	3.97 ± 0.05	5.40 ± 0.04	5.71 ± 0.02
Sun98‐H2	7.05 ± 0.08	7.85 ± 0.15	5.67 ± 0.17	6.97 ± 0.23	4.65 ± 0.34	4.68 ± 0.07	3.57 ± 0.08	3.41 ± 0.04
Sun98‐H3	6.52 ± 0.03	7.46 ± 0.12	6.63 ± 0.15	7.43 ± 0.12	4.80 ± 0.27	4.76 ± 0.05	3.80 ± 0.08	3.95 ± 0.15
Sun98‐H4	6.34 ± 0.06	6.02 ± 0.06	5.66 ± 0.25	5.94 ± 0.16	5.74 ± 0.30	5.57 ± 0.07	6.47 ± 0.03	6.50 ± 0.10
Sun98‐H5	6.61 ± 0.05	6.12 ± 0.06	5.51 ± 0.12	6.21 ± 0.07	5.95 ± 0.07	6.46 ± 0.09	4.38 ± 0.07	4.24 ± 0.01
Sun98‐H6	6.82 ± 0.10	8.76 ± 0.06	6.50 ± 0.12	6.53 ± 0.17	5.12 ± 0.05	4.49 ± 0.06	4.97 ± 0.00	4.73 ± 0.05
Sun98‐H7	5.49 ± 0.11	7.16 ± 0.09	5.51 ± 0.20	5.96 ± 0.15	6.29 ± 0.07	4.84 ± 0.06	5.53 ± 0.04	6.04 ± 0.16
Sun98‐H8	6.36 ± 0.05	7.37 ± 0.07	6.20 ± 0.09	6.36 ± 0.12	4.61 ± 0.03	4.16 ± 0.07	4.48 ± 0.02	4.05 ± 0.11
Sun98‐H9	5.95 ± 0.04	6.15 ± 0.10	6.14 ± 0.12	5.45 ± 0.06	5.47 ± 0.05	5.63 ± 0.12	6.54 ± 0.11	5.74 ± 0.09
Sun98‐H10	6.02 ± 0.06	6.97 ± 0.32	5.85 ± 0.34	7.26 ± 0.12	5.87 ± 0.05	4.87 ± 0.08	5.21 ± 0.01	5.43 ± 0.02
Sun98‐H11	6.18 ± 0.04	6.60 ± 0.06	5.90 ± 0.06	6.48 ± 0.07	5.22 ± 0.06	3.59 ± 0.06	6.65 ± 0.03	6.87 ± 0.03
Sun98‐H12	6.00 ± 0.09	8.32 ± 0.02	5.28 ± 0.37	5.52 ± 0.09	5.49 ± 0.05	6.08 ± 0.03	6.54 ± 0.01	6.44 ± 0.02
Ghasem	6.04 ± 0.05	6.44 ± 0.06	6.19 ± 0.09	5.31 ± 0.09	6.34 ± 0.01	3.25 ± 0.05	6.02 ± 0.05	6.51 ± 0.07
Mean	6.20	7.09	5.85	6.27	5.46	4.79	5.35	5.35
LSD1%	0.25	0.48	0.78	0.53	0.58	0.21	0.22	0.32
C (%)	14.4		7.2		−12.3		0.0	

*Note*: Values are means ± standard error.

Abbreviation: C, Changes under stressed condition in compared with normal irrigation.

Despite palmitic acid, the mean value for stearic acid content was decreased by 0.66% under drought stress in 2019 while unchanged in 2020 (Table 5). The highest stearic acid content was recorded for Sun98‐H11 under stressed (6.87%) and normal (6.65%) irrigations in 2020. The check hybrid Ghasem had the lowest stearic acid content under drought stress condition (3.25%) in 2019 and was affected more than other hybrids by drought stress (3.09% reduction). Amount of this fatty acid was stable for Sun98‐H2 and Sun98‐H3 in 2019 and for Sun98‐H4 in 2020.

Oleic acid content was decreased under drought stress at a rate of 2.5% in both years (Table 6). Sun98‐H1 expressed a considerably higher content of oleic acid under normal (33.72 and 26.87% in 2019 and 2020, respectively) and stressed conditions (28.09 and 25.29% in 2019 and 2020, respectively); however, Sun98‐H4 had the highest (26.91%) oleic acid content under optimum irrigation in 2020. The lowest oleic acid content was observed in Sun98‐H3 under normal and stressed irrigation in both years. The highest reduction was recorded for Sun98‐H6 in 2019 (6.15%) and Sun98‐H2 (5.77%) in 2020. The amount of oleic acid in Sun98‐H4 was stable under normal and stressed conditions in 2019.

**TABLE 6:** Comparison of sunflower hybrids for oleic and linoleic acids contents (%) under normal (N) and drought stress (S) conditions.

Hybrids	Oleic acid				Linoleic acid			
	2019		2020		2019		2020	
	N	S	N	S	N	S	N	S
Sun98‐H1	33.72 ± 0.29	28.09 ± 0.23	26.87 ± 0.38	25.29 ± 0.59	54.05 ± 0.36	60.62 ± 0.28	61.15 ± 0.33	60.70 ± 0.71
Sun98‐H2	17.53 ± 0.42	16.08 ± 0.34	22.19 ± 0.52	16.42 ± 0.46	69.37 ± 0.69	70.85 ± 0.40	67.16 ± 0.60	71.80 ± 0.64
Sun98‐H3	15.87 ± 0.42	15.21 ± 0.56	18.82 ± 0.49	16.04 ± 0.26	71.23 ± 0.70	71.50 ± 0.69	69.50 ± 0.34	70.98 ± 0.37
Sun98‐H4	17.94 ± 0.35	18.04 ± 0.12	26.91 ± 0.44	24.88 ± 0.56	68.54 ± 0.66	68.83 ± 0.23	59.68 ± 0.35	61.03 ± 0.44
Sun98‐H5	19.87 ± 0.29	19.02 ± 0.22	20.97 ± 0.33	21.22 ± 0.32	65.69 ± 0.41	66.76 ± 0.26	67.65 ± 0.16	66.90 ± 0.50
Sun98‐H6	18.94 ± 0.39	12.79 ± 0.68	24.43 ± 0.52	21.52 ± 0.74	68.59 ± 0.42	73.35 ± 0.80	62.93 ± 0.34	65.77 ± 0.55
Sun98‐H7	25.44 ± 0.49	23.27 ± 0.20	24.02 ± 0.34	23.55 ± 2.22	61.17 ± 0.53	64.27 ± 0.34	63.59 ± 0.18	62.85 ± 2.00
Sun98‐H8	19.88 ± 0.38	19.10 ± 0.29	20.28 ± 0.57	19.13 ± 0.63	67.69 ± 0.50	68.87 ± 0.39	67.90 ± 0.43	69.37 ± 1.18
Sun98‐H9	17.29 ± 0.58	15.80 ± 0.39	22.05 ± 0.51	16.34 ± 0.59	70.00 ± 0.58	70.36 ± 0.59	65.64 ± 1.91	70.85 ± 0.58
Sun98‐H10	20.32 ± 0.57	17.25 ± 0.28	20.43 ± 0.59	18.48 ± 0.45	66.10 ± 0.63	69.24 ± 0.34	66.97 ± 0.73	67.52 ± 0.54
Sun98‐H11	19.88 ± 0.29	18.16 ± 0.10	23.78 ± 0.45	21.87 ± 0.42	66.77 ± 0.43	70.95 ± 0.41	61.87 ± 0.56	62.65 ± 0.53
Sun98‐H12	20.36 ± 0.10	17.19 ± 0.49	23.03 ± 0.48	21.05 ± 0.28	66.60 ± 0.19	66.00 ± 0.58	63.69 ± 0.51	65.44 ± 0.32
Ghasem	23.17 ± 0.42	18.05 ± 0.22	23.23 ± 0.42	19.20 ± 0.15	62.67 ± 0.43	71.74 ± 0.37	63.04 ± 0.46	67.18 ± 0.13
Mean	20.79	18.31	22.85	20.38	66.04	68.72	64.68	66.39
LSD 1%	1.28	1.03	1.65	3.15	1.62	1.22	2.72	3.24
C (%)	−11.9		−10.8		4.1		2.4	

*Note*: Values are means ± Standard error.

Abbreviation: C, Changes under stressed condition compared with normal irrigation.

Despite oleic acid, the mean of linoleic acid content was increased under drought stress in both years (2.68 and 1.71% in 2019 and 2020, respectively). There were differentiated responses between the hybrids. For example, Sun98‐H12 in 2019 experienced a reduction of this fatty acid under stressed irrigation (Table 6). The highest content of linoleic acid was recorded for Sun98‐H6 (73.35%) under drought stress condition in 2019. Sun98‐H1 had the lowest linoleic acid content under normal (54.05%) and drought stress conditions (60.62%) in 2019. In 2020, Sun98‐H4 had the lowest content of this fatty acid (59.68%) under normal irrigation and Sun98‐H1 under stressed condition (60.70). The highest increase in linoleic acid content was recorded for Sun98‐H1 (6.57%) in 2019 and for Sun98‐H9 (5.20%) in 2020. The content of this fatty acid was stable in Sun98‐H3 in 2019 and Sun98‐H1 in 2020 (Table 6).

### 3.4 General effect of drought stress on agronomic traits and oil characteristics

**FIGURE 2:**
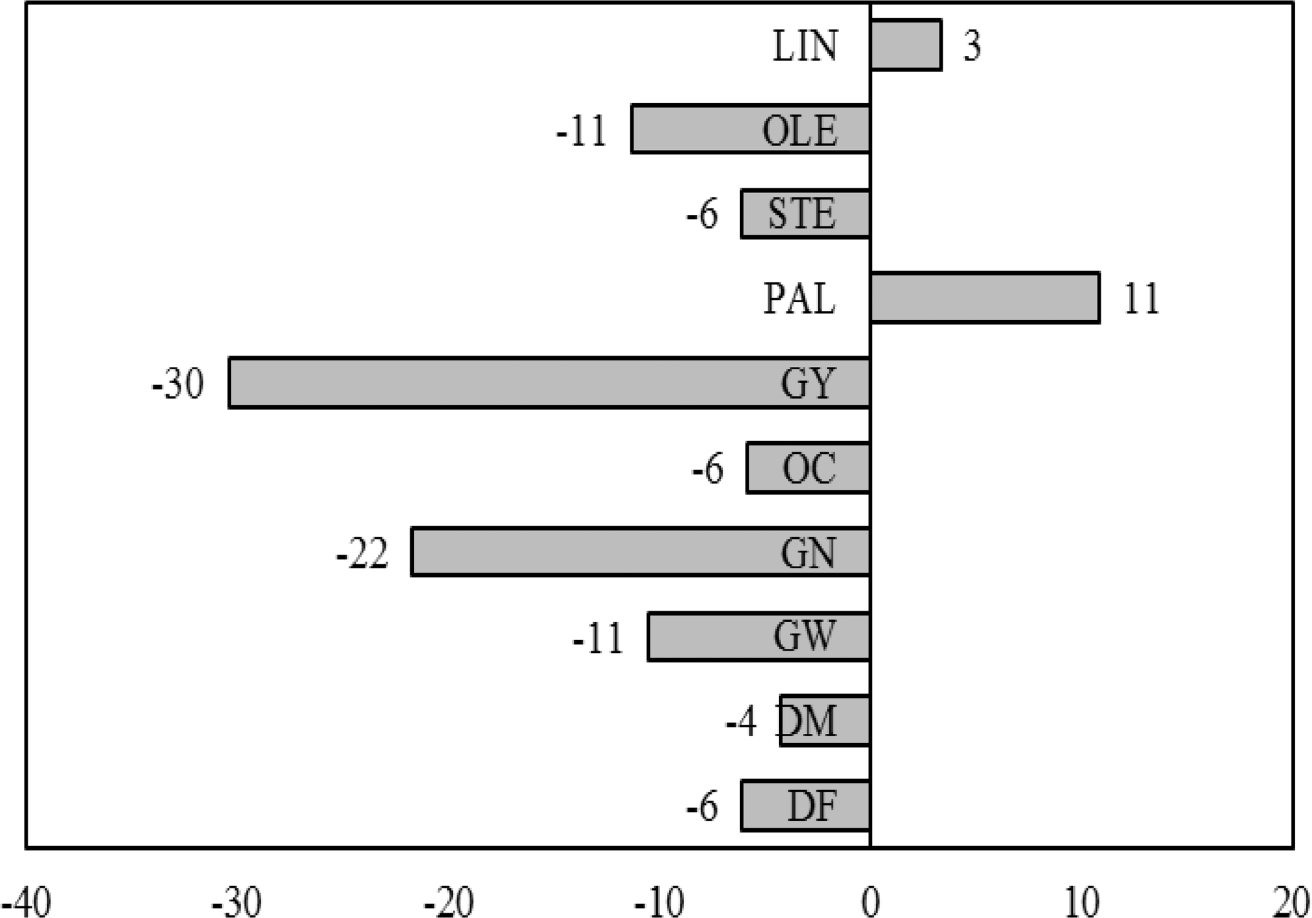
Effect of drought stress on agronomic traits and fatty acid composition of sunflower oil under normal and drought stress conditions. The values for each trait were computed according to the mean values of three replications during the 2 years of study (2019 and 2020) in Karaj, Iran. The negative values denote the reduction while positive ones to the increasing of mean value for the related trait under drought stress in compared with normal irrigation. DF, days to flowering; DM, days to physiological maturity; GN, grain number per head; GW, 1000 grains weight; GY, grain yield; LIN, linoleic acids; OC, oil content; OLE, oleic acid; PAL, palmitic acid; STE, stearic acid.

Drought stress caused a reduction in days to flowering (6%) and physiological maturity (4%) as well in 1000‐grains weight (11%), grain numbers per head (22%), oil content (6%), and grain yield (30%) (Figure 2). Among the fatty acids, palmitic and linoleic acids contents were increased (11 and 3% respectively), while stearic and oleic acid contents were decreased (6 and 11%). There were different responses of the hybrids in terms of fatty acids to the drought stress in this study. The contents of palmitic and linoleic acids were increased, while the content of stearic and oleic acids decreased under drought stress. The most increases were recorded for palmitic acid (11%) and the most reduction was recorded for oleic acid (11%).

### 3.5 Drought tolerance features

**FIGURE 3:**
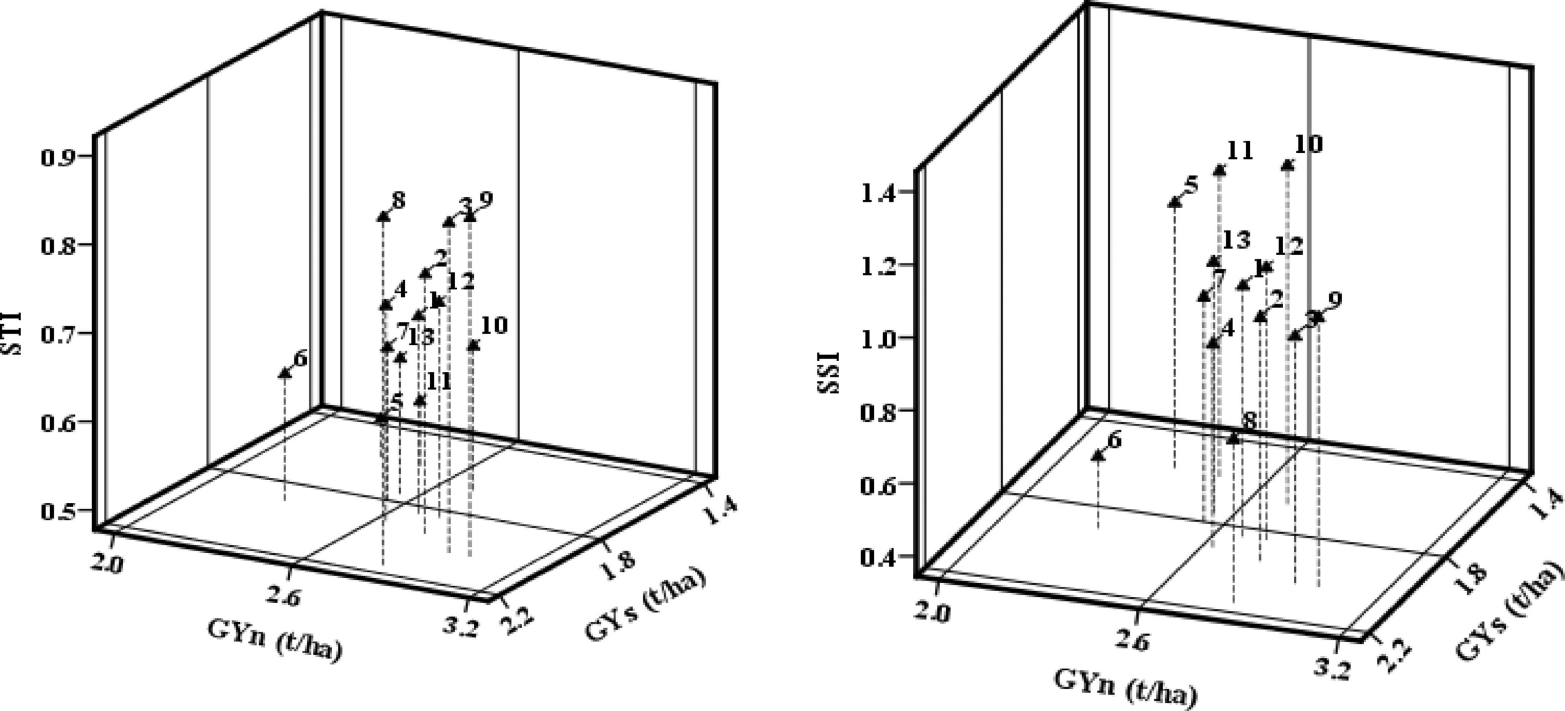
Three‐dimensional plot of grain yield (t/ha) under normal (GYn) and drought stress conditions (GYs) with Stress Tolerance Index (STI) (left) and Stress Susceptibility Index (SSI) (right). Each triangle with related dashed line shows the position of each hybrid. The names of the hybrids are shown in Table 2. The means of 2 years data for grain yield of each hybrid in three replications were used to estimation of STI and SSI.

The results expressed Sun 98‐H8, Sun 98‐H3, and Sun 98‐H9 as drought‐tolerant hybrid with highest STI (Table 7). These hybrids had the highest grain yield under normal (2.8–3.0 t/ha) and stressed conditions (2.0–2.1 t/ha) located in region A based on Fernandez (1992) groups (Figure 3); between them, Sun98‐H8 experienced the lowest reduction (24%) in grain yield by drought stress and had the highest STI (0.87) and a relative low SSI (0.79). Sun98‐H6 had the lowest reduction in grain yield (16%) under stressed condition and was located in region C. Sun98‐H10 was affected more than other hybrids by drought stress with a 38% reduction of grain yield showing the highest SSI (1.27) located in region B. The lowest grain yield recorded for Sun98‐H5 and Sun98‐H11 (1.6 t/ha) under stressed condition caused locating them in region D as the low‐yielding hybrids under normal and stressed conditions.

**TABLE 7:** Drought tolerance indices for sunflower hybrids according to the 2 years data (2019 and 2020).

No.	Hybrids	GYn (t/ha)	GYs (t/ha)	R (%)	STI	SSI
1	Sun98‐H1	2.68	1.84	31.24	0.70	1.03
2	Sun98‐H2	2.79	1.94	30.64	0.77	1.01
3	Sun98‐H3	2.94	2.02	31.05	0.85	1.02
4	Sun98‐H4	2.63	1.91	27.23	0.72	0.90
5	Sun98‐H5	2.32	1.57	32.41	0.52	1.07
6	Sun98‐H6	2.28	1.90	16.38	0.62	0.54
7	Sun98‐H7	2.54	1.80	29.15	0.65	0.96
8	Sun98‐H8	2.83	2.15	23.90	0.87	0.79
9	Sun98‐H9	3.01	2.02	32.92	0.86	1.08
10	Sun98‐H10	2.71	1.66	38.48	0.64	1.27
11	Sun98‐H11	2.46	1.58	35.99	0.55	1.18
12	Sun98‐H12	2.75	1.84	33.05	0.72	1.09
13	Ghasem	2.54	1.75	31.21	0.63	1.03
	Mean	2.65	1.85	30.41	0.70	1.00

*Note*: The means of 2 years data for grain yield of each hybrid were used to estimation of R, STI and SSI.

Abbreviations: GYs and GYs, Grain yield under normal and stressed conditions respectively; R, Reduction rate of grain yield under drought stress condition; SSI, Stress susceptibility index; STI, Stress tolerance index.

### 3.6 Relationships between the grain yield, agronomic traits, and oil characteristics

Grain numbers/head had a higher correlation with grain yield (0.621 and 0.537 under normal and stressed conditions, respectively). The correlations between grain numbers and grain weigh were negative in both conditions (Table 8). All the correlation coefficients between the fatty acids were significant in both conditions except for stearic and oleic acids under stressed condition. Correlation coefficients of palmitic and linoleic acids with stearic and oleic acids were negative, while all of the others were positive in both conditions.

**TABLE 8:** Correlation coefficients between agronomic characteristics and fatty acid components of sunflower under normal (a) and drought stress (b) conditions.

Traits	DF	DM	GW	GN	OC	GY	PAL	STE	OLE
(a)									
DM	0.408**								
GW	−0.028ns	0.124ns							
GN	0.214ns	0.068ns	−0.574**						
OC	0.257*	0.100ns	0.074ns	−0.106ns					
GY	0.192ns	0.157ns	0.259**	0.621**	−0.049ns				
PAL	−0.194ns	−0.098ns	−0.473**	0.219ns	−0.151ns	−0.186ns			
STE	−0.120ns	−0.414**	0.072ns	−0.316**	−0.202ns	−0.265*	−0.250*		
OLE	0.119ns	0.218ns	0.392**	−0.448**	0.222ns	−0.165	−0.632**	0.337**	
LIN	−0.031ns	−0.107ns	−0.354**	0.506**	−0.159ns	0.256*	0.551**	−0.513**	−0.958**
(b)									
DM	0.179ns								
GW	−0.319**	−0.043ns							
GN	0.462**	0.227*	−0.596**						
OC	−0.291**	0.177ns	0.116ns	−0.186ns					
GY	0.213ns	0.227*	0.316**	0.537**	−0.010ns				
PAL	0.161ns	0.107ns	−0.302**	0.331**	−0.333**	0.019ns			
STE	−0.110ns	−0.126ns	0.286*	−0.341**	−0.044ns	−0.142ns	−0.399**		
OLE	−0.227*	0.203ns	0.352**	−0.379**	0.188ns	−0.117ns	−0.389**	0.219ns	
LIN	0.220ns	−0.162ns	−0.389**	0.416**	−0.098ns	0.149ns	0.306**	−0.501**	−0.928**

*Note*: ns is insignificant and * and ** are significant at statistical probability *p* < .05 and *p* < .01 respectively. The means of 2 years data that recorded on 13 sunflower hybrids were used to estimation of correlation coefficients.

Abbreviations: DF, days to flowering; DM, days to maturity; GN, grain numbers/head; GW, 1000‐grains weight; GY; grain yield; LIN, linoleic acids; OC; oil content; OLE, oleic acid; PAL, palmitic acid; STE, stearic acid.

**FIGURE 4:**
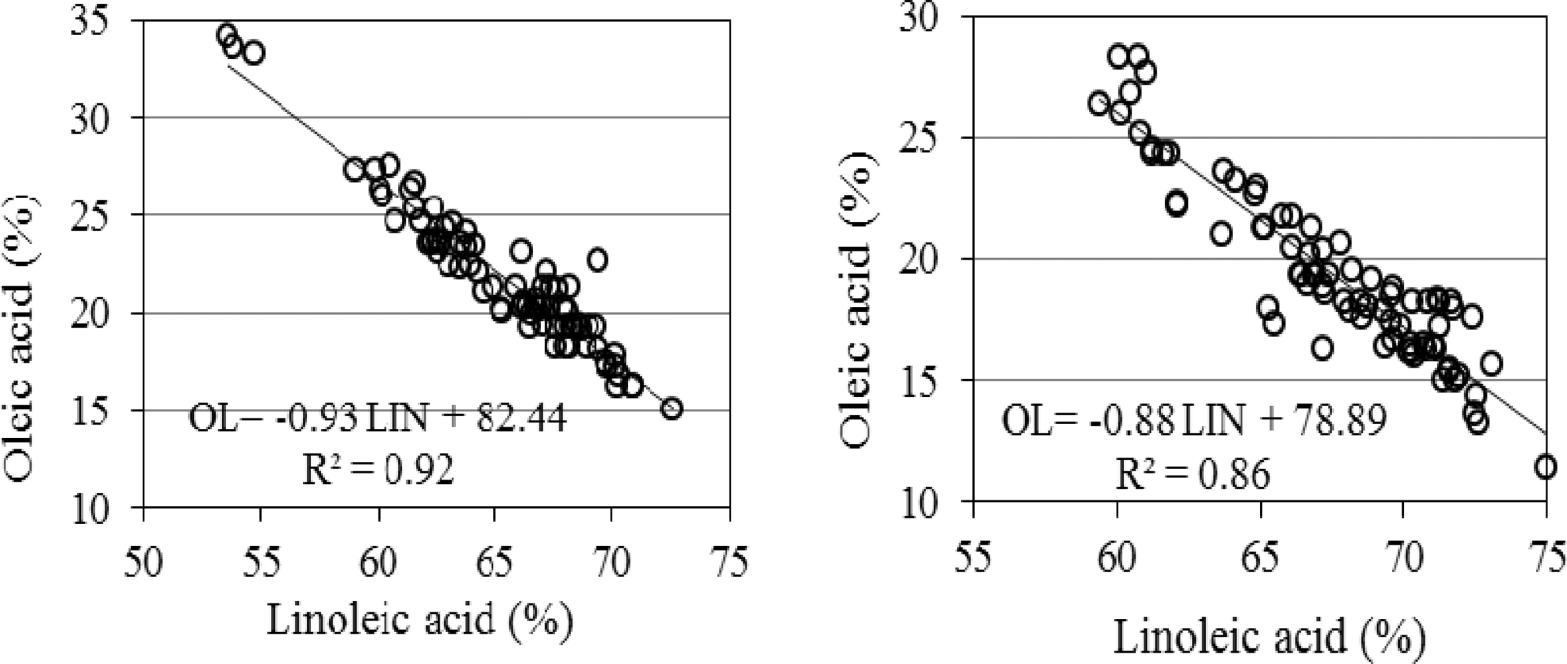
Relationship between oleic and linoleic acids in sunflower oil under normal (left) and drought stress (right) conditions. The values for fatty acid contents are ratio of each fatty acid to total fatty acids in sunflower grains oil measured by a gas chromatography instrument. All data from three replications in each year were used to fit the curves under normal and drought stress conditions. The circles represent the value of each hybrid. OL, Oleic acid; LIN, Linoleic acid.

According to the fitted line by regression analysis, there was a negative linear relationship between oleic and linoleic acid contents in both normal and stressed conditions. However, more part of oleic acid variation was explained by linoleic acid variation under optimum irrigation (Figure 4). Oleic acid content decreased as linoleic acid content increased in both normal and drought stress conditions. Increasing each percent of oleic acid led to about 0.93% and 0.88% decrease in linoleic acid content in normal and drought stress conditions respectively.

**FIGURE 5:**
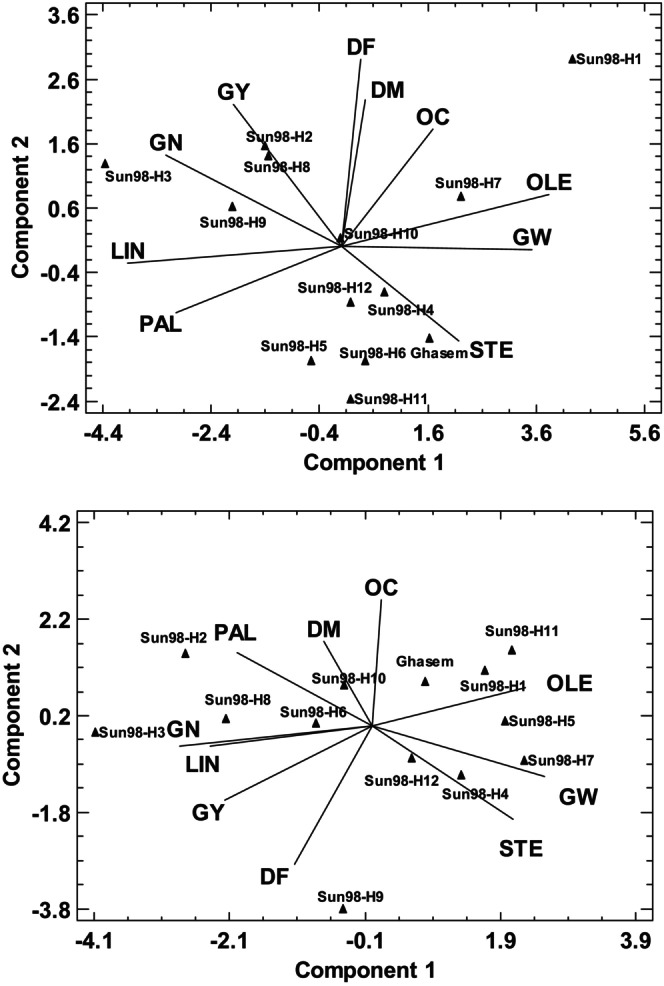
The biplot of the principal component analysis representing the relationships between the agronomic traits and major fatty acids of sunflower under normal (up) and drought stress conditions (down). The triangles represent the position of the hybrids in relation to the related vectors of the traits (solid lines). The values for each trait are mean values of three replications during the 2 years of study (2019 and 2020). DF, days to flowering; DM, days to physiological maturity; GN, grain number per head; GW, 1000 grains weight; GY, grain yield; LIN, Linoleic acids; OC, oil content; OLE, oleic acid; PAL, palmitic acid; STE, stearic acid.

Principal component analysis revealed the associations between the traits and the hybrids and classified the hybrids according to the related characteristics (Figure 5). The first principal component (PC1) was affected by linoleic and oleic acids, followed by grains weight and grain numbers/head under normal condition. The effects of oleic acid and grains weight were positive, while the others were negative. The second principal component (PC2) was affected mainly by phenological traits, followed by grain yield and oil content; all were positive. Under stressed condition, there were relatively similar status for PC1 but PC2 was more affected by flowering time and oil content. Regarding close angle of the related vectors, grain numbers/head had higher correlation with grain yield than grains weight under normal and stressed conditions. The PCA determined positive correlations between linoleic and palmitic acid contents and between oleic and stearic acid contents. Negative correlations between linoleic/palmitic acid contents with oleic/stearic acid contents were also determined in both conditions. There was a considerable relationship between linoleic and palmitic acids under normal condition.

Sun98‐H3, Sun98‐H9, Sun98‐H2, and Sun98‐H8 were characterized in PCA biplot by higher grain yield, grain numbers/head, and higher linoleic and palmitic acid contents, while Sun98‐H6, Sun98‐H11, Ghasem, etc. were classified in the reverse side as the low‐yielding hybrids with higher stearic acid content under normal irrigation. In addition, Sun98‐H3, Sun98‐H2, and Sun98‐H8 expressed as high‐yielding hybrids under stressed condition. Sun98‐H11, Sun98‐H5, and Sun98‐H7 were differentiated with lower grain yields but higher oleic acid content under drought stress condition.

## 4 DISCUSSION

### 4.1 Effect of drought stress on agronomic features

The results of this study showed that sunflower hybrids adjust their phenology differentially under drought stress. The difference between the hybrids under normal or stressed conditions was not considerable due to the implying of early maturity as one of the main breeding objectives in selection process of these hybrids. Goyne et al. (1982) also reported little genetic variability in the phenology of commercial sunflower cultivars due to the importance of early maturity in breeding programs. Early flowering/maturity is an escape strategy that enables the genotypes to reproduce before the onset of severe stress successfully (Bacelar et al., 2012). It should be noted that early flowering could affect grain yield and oil content negatively (De la Vega & Chapman, 2010). Therefore, a balance must be struck between flowering and maturity time with grain yield. With the development of drought in Iran, sunflower cultivation has shifted to marginal lands or secondary cultivation recently so breeding programs have focused on improving the early mature hybrids. Consequently, there was a narrow range of variability between the hybrids for phenological characteristics.

The responses of the hybrids to drought stress in terms of grain yield within and between the years were variable at a range of 16%–38% that reflect stability/instability of the hybrids between the watering regimes and the 2 years of study. Generally, mean of grain yield for sunflower hybrids were decreased 30% by drought stress (30%) in this study and grain numbers per head was affected more than 1000‐grains weight. Negative effect of drought stress on sunflower grain yield has been reported in numerous studies (Akcay & Dagdelen, 2016; Chimenti et al., 2002; Ghaffari et al., 2012; Todorovic et al., 2009). Due to the higher impact of grain number on grain yield of sunflower (Shankar et al., 2006), it is concluded that the genotypes with higher ability in maintaining higher grain number can produce higher grain yield under drought stress. The response of the hybrids to the years was variable in terms of 1000‐grains weight and grain numbers/head, which could be assumed as the source of grain yield instability over the years. In 2019, higher temperature in monthly average could negatively affect seed weight by shortening the grain filling stage, explaining higher grain numbers/head. Regarding the reduction of grain numbers in 2020 as the main determinant of sunflower yield (Ghaffari et al., 2019; Miller & Fick, 1997), increased yield in 2020 indicated that sunflower could compensate for grain yield by increasing of grains weight. Changes in climatological parameters as higher humidity in 2020 could be a cause for reduction of grain yield in this study that is in accordance with the reports of De la Vega et al. (2001). Grain yield is determined mainly by genetic factors. However, identifying the environmental parameters responsible for the preservation of grain yield can help the improvement of more stable sunflower hybrids under different environmental stresses.

The multi‐variate PCA method revealed a clear structure between the data. This method was used to determine the structure between the genotypes and environments (De La Vega et al., 2001) and heterosis (Zakeri Haddadan et al., 2020). In accordance with Skoric (2012), the high‐yielding hybrids had lower oleic acid content, and grain numbers/head was expressed as the main determinant of grain yield that is in accordance with Shankar et al. (2006). The higher potential of early flowering hybrids for grain yield under stressed condition attributed to their escape ability from higher temperatures during the flowering time, which limits pollinators needed for the fertilization process. The PCA method revealed that a higher potential of sunflower grain yield could be achieved by standard oil sunflower hybrids and an increased area of cultivation of high oleic sunflower may reduce oil production considerably. Therefore, the decision on which type of sunflower to grow with oil quality aspects needs to comprehensive considerations.

### 4.2 Effect of drought stress on oil characteristics of sunflower

Drought stress reduced oil content in this study, which is in accordance with other reports (Akcay & Dagdelen, 2016; Ghaffari et al., 2012; Todorovic et al., 2009). Variability of oil content during the 2 years and differential response of the hybrids to the drought stress imply to the fact that genetic and environmental factors control sunflower oil content as also reported by Connor and Hall (1997). Evidence shows that climatic variables are the most important causes of the hybrid by sowing date interaction for oil content in sunflower (Balalic et al., 2012). There is evidence that shows sunflower oil quantity and quality is affected by fluctuations in temperature, solar radiation, and moisture availability in different years (Hassan et al., 2011).

The contents of palmitic, linoleic, and linolenic acids were increased, while the contents of stearic, oleic, and behnic acids were decreased in response to drought stress in this study (Figure S1). These are in accordance with Petcu et al. (2001) and Popa et al. (2017) but contradictory to the reports of Flagella et al. (2002). The physiological bases of how environmental factors affect oil quality have been studied (Connor & Hall, 1997) but have not been integrated to evaluate the effect of these changes on grain yield. Fatty acid metabolic pathways play a significant role in the biosynthesis of precursors for cuticular components and have a major role in plant responses to abiotic stresses (Kachroo & Kachroo, 2009). Enhancing drought tolerance in transgenic plants by increasing cuticular wax and thereby improving the water‐retaining capacity has also been reported (Zhang et al., 2005). Reducing oleic acid content by reducing stearoyl desaturase activity to maintain a higher level of stearic acid content may be an adaptability response to maintain of membrane integrity and fluidity (Vannini et al., 2004) and can help the preservation of sunflower yield under drought stress condition. The critical role of cell‐structure integrity has been reported as one of the major aspects of drought tolerance in sunflower (Ghaffari et al., 2017).

The different responses of the hybrids to the years in terms of palmitic acid content may be attributed to the differential sensitivity of the responsible denatures enzymes in the fatty acid biosynthesis chain (Lagravere et al., 2000). These fatty acids make acylation of the proteins those are important for the normal function of proteins by anchoring in membranes or folding them (Gurr & Harwood, 1991). Activation of antioxidant‐related genes can protect fatty acids against oxidative stress and finally affect their stability (Semchuk et al., 2009). The concentration of saturated fatty acids, mainly palmitic acid, cannot fall below a certain level for operating normal function of the lipid bilayer of membranes (Zhukov, 2015). Differentiate changes in palmitic acid content may associate with different capacities of the hybrids in palmitic acid‐related protective proteins under the drought stress condition in this study. Palmitic acid is undesirable for human consumption mainly due to the rising of serum cholesterol levels, while oils rich in oleic acid are preferred as it combines the hypo‐cholesterolemic effect (Mensink & Katan, 1989) and a greater oxidative stability than linoleic acid (Smith et al., 2007). Longer chain fatty acids such as stearic acid could serve as substrates for synthesizing biologically active molecules as cuticles that can reduce this fatty acid in response to drought stress (Millar & Kunst, 1997). It has been reported that the early steps of stearic acid elongation in epidermal cells may be indispensable for plant growth (Farmer et al., 1998). Increased levels of stearic acid in some of the hybrids could be associated with pathogenic defense (Kachroo et al., 2008).

A strong negative correlation between oleic and linoleic acids was confirmed by regression and principal component analysis which is in accordance with (Popa et al., 2017; Sobrino et al., 2003). All of the fatty acids are biosynthesized in a common chain, so the content of them relates to each other. Palmitic acid is the first one in the biosynthetic chain of fatty acids in sunflower and the others are synthesized by adding of carbon atoms as stearic acid formation, which follows the palmitic acid, and so on, oleic acid is formed through the desaturation of stearic acid (Baldini et al., 2000). The concentration of linoleic acid increases until the end of grain maturation whereas the content of oleic acid decreases due to the desaturation of oleic acid to linoleic acid (Skoric, 1992). This may explain the insignificant correlation of stearic acid with oleic acid content under stressed condition. More involvement of stearic acid in signaling/pathogenic defense (Kachroo et al., 2008) could be a cause for limiting oleic acid biosynthesis under stressed condition. Unlike the normal condition, none of the fatty acids had not significant correlation with grain yield. The fatty acid biosynthesis chain probably works in optimum conditions, but the abnormal associations were expressed with increased demand for stearic acid under drought conditions.

The results of this study demonstrated that drought stress reduce oil quality of sunflower in terms of oil stability by reduction of oleic acid content and increased level of saturated fatty acids. With the development of mid and high oleic acid sunflower hybrids, genetic drift, and transferring the responsible genes to standard sunflowers could enhance the effect of environmental effects on sunflower yield on a large scale.

## 5 CONCLUSIONS

Phenology of plants, including sunflower, is the most important feature that is affected by drought stress. In this study, drought stress accelerated flowering and maturity time of sunflower hybrids which caused other effects in gain yield and its components. Drought stress caused a reduction in 1000‐grains weight (11%), grain numbers per head (22%), and oil content (6%) compared with normal irrigation, so among the grain yield components, grain number was affected more than two other components. The simultaneous decrease in the weight of 1000 grains and the number of grains per pod was accompanied by a sharp decrease in seed yield (30% in average). Despite the overall negative effect of drought stress on the agronomic characteristics of sunflower, it was observed that both different traits and hybrids are differentially affected by drought stress. For example, the range of effect of drought stress on the grain yield of the hybrids was 16%–38%. This makes the issue of selection criteria important in breeding programs in order to improve sunflower hybrid cultivars under drought stress conditions. Considering that the number of grains per head was affected by drought stress more than other yield components, therefore, the selection of genotypes in terms of more grain number under drought stress condition can lead to the identification of drought tolerant sunflower hybrids. Among the hybrids, Sun98‐H8 was differentiated as the most drought tolerant and Sun98‐H10 as the most drought‐sensitive hybrids in this study which differentiated with a relative higher grain number per head respectively. Although Sun98‐H3 had the highest grain number in both years (1076 and 1064) but due to the very low 1000 grains weight (42 and 36 g in 2019 and 2020 respectively) experienced a higher reduction of grain yield (31%) than Sun98‐H8 (23%). So according to the results of this study, grain number per head was the most important determinant of grain yield under drought stress condition.

Considering the fatty acids, among the fatty acids, the contents of palmitic and linoleic acids were increased (11 and 3%, respectively) while stearic and oleic acids were decreased (6 and 11%) reduced under drought stress. Oleic acid is the most preferred fatty acid of sunflower because of its association with higher stability to heat oxidation, long shelf life, lowering cholesterol, and heart disease (Wardlaw & Snook, 1990). The results demonstrated that drought stress reduce oil quality of sunflower by reduction of oleic acid content and increased level of saturated fatty acids. This can also reduce the stability of sunflower oils extracted from the grains that harvested from drought stressed fields. Considering the doubts about the adverse effects of high linoleic acid consumption in the diet (Jandacek, 2017) and the general agreement on the usefulness of oleic acid in another hand, a decrease in oleic acid content under drought stress causes reduction in quality of sunflower oil produced under drought stress conditions in terms of nutritional value and oil stability.

The hybrids with higher grain yield under drought stress condition expressed lower oleic acid content than low‐yielding hybrids, but this was not expressed in correlation coefficients, which could be due to the expression of this statue in a lower set of thy hybrids. The changes in fatty acids may be an adaptability response to maintaining membrane integrity and fluidity that can enhance the preservation of sunflower yield under drought stress condition. The results indicated that a higher potential of sunflower grain yield could be achieved by standard oil sunflower hybrids, and increased development of high oleic hybrids could lead to a reduction in sunflower production on a large scale. Integrating the information on the effect of fatty acid changes on grain yield can provide a clear picture of the response of sunflower genotypes to drought stress.

## AUTHOR CONTRIBUTIONS

**Mehdi Ghaffari:** Conceptualization (lead); data curation (lead); formal analysis (lead); investigation (lead); methodology (lead); project administration (lead); resources (lead); software (lead); supervision (lead); writing – original draft (lead); writing – review and editing (lead). **Amir Gholizadeh:** Data curation (supporting); investigation (supporting); methodology (supporting); software (supporting); writing – original draft (supporting); writing – review and editing (supporting). **Saeed Rauf:** Formal analysis (supporting); investigation (supporting); writing – original draft (supporting); writing – review and editing (supporting). **Farnaz Shariati:** Data curation (equal); methodology (supporting); software (supporting).

## FUNDING INFORMATION

This research received no specific grant from any funding agency in the public, commercial, or not‐for‐profit sectors.

## CONFLICT OF INTEREST STATEMENT

The authors have no conflicts of interest to declare that they are relevant to the content of this article.

### ETHICS STATEMENT

This study does not involve any human or animal testing.

## INFORMED CONSENT

Written informed consent was obtained from all study participants.

## Supporting information


Appendix S1.

## Data Availability

The data that support the findings of this study are available from the corresponding author upon reasonable request.
