# Supplementary material for: Drought‐stress induced changes of fatty acid composition affecting sunflower grain yield and oil quality
Source: Food Sci Nutr. 2023 Sep 19;11(12):7718–31. doi: 10.1002/fsn3.3690 (PMC10724631; doi:10.1002/fsn3.3690)
Supplement: Supplementary file 1 — Appendix S1. [file FSN3-11-7718-s001.doc]

| **Supplemental TABLE 1** The list and pedigree of the hybrids evaluated under normal and drought stress condition during two years (2019 and 2020) in Karaj, Iran | | | |
| --- | --- | --- | --- |
| No. | Hybrid | Pedigree | Origin |
| 1 | Sun98-H1 | R131 x AGK38 | SPII, Iran |
| 2 | Sun98-H2 | R131 x AF81-222 | SPII, Iran |
| 3 | Sun98-H3 | R131 x AGK32 | SPII, Iran |
| 4 | Sun98-H4 | RGK33 x AGK38 | SPII, Iran |
| 5 | Sun98-H5 | RGK33 x AF81-222 | SPII, Iran |
| 6 | Sun98-H6 | RGK33 x AGK32 | SPII, Iran |
| 7 | Sun98-H7 | RGK15 x AGK38 | SPII, Iran |
| 8 | Sun98-H8 | RGK15 x AF81-222 | SPII, Iran |
| 9 | Sun98-H9 | RGK15 x AGK32 | SPII, Iran |
| 10 | Sun98-H10 | RF81-82 x AGK38 | SPII, Iran |
| 11 | Sun98-H11 | RF81-82 x AF81-222 | SPII, Iran |
| 12 | Sun98-H12 | RF81-82 x AGK32 | SPII, Iran |
| 13 | Ghasem | R14 x A1221 | SPII, Iran |
| SPII; Seed and Plant Improvement Institute | | | |

| 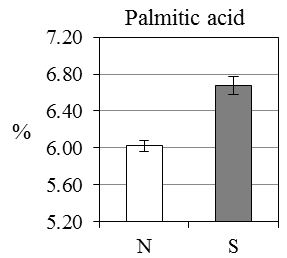 | 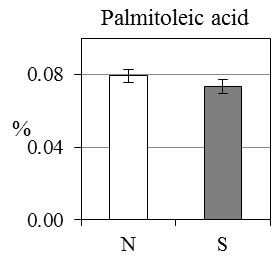 | 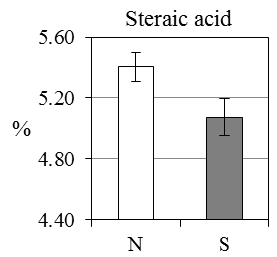 |
| --- | --- | --- |
| 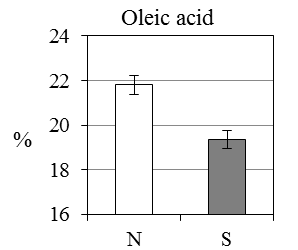 | 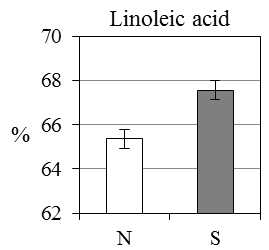 | 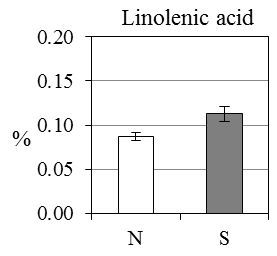 |
| 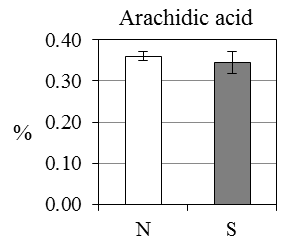 | 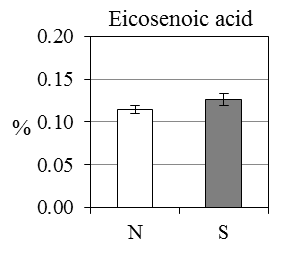 | 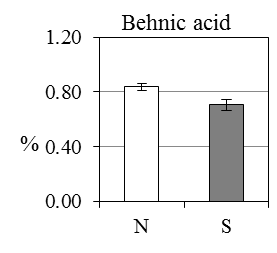 |
| 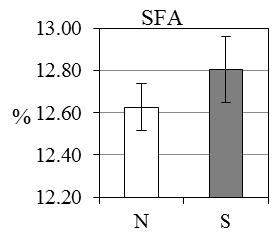 | 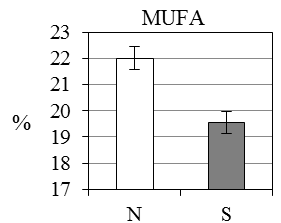 | 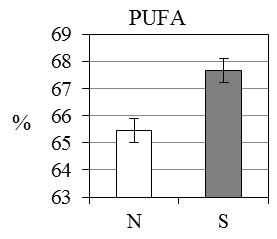 |
| **Supplemental FIGURE 1** Comparison of mean values for sunflower fatty acids under normal (N) and drought stress (S) conditions. Each figure represents the content (%) of related fatty acid mentioned above the graph. The values for the fatty acids are mean values that recorded on 13 sunflower hybrids in three replications during the two years of study (2019 and 2020) under normal (white bars) and drought stress (grey bars) conditions. Bars are means ± 1 standard error. SFA, MUFA and PUFA denote to the saturated, mono-unsaturated and poly unsaturated fatty acids respectively. | | |
